# Emotional and Clinical Challenges While Dealing With a Blind-Deaf-Mute Patient

**DOI:** 10.7759/cureus.9780

**Published:** 2020-08-16

**Authors:** Amr Elmoheen, Waleed Salem, Mohamed Yousry, Khalid Bashir

**Affiliations:** 1 Emergency Medicine, Hamad Medical Corporation, Doha, QAT; 2 Qatar University Health, College of Medicine, Doha, QAT

**Keywords:** blind, deaf, mute, emotional, communication, emergency medicine, clinical challenge

## Abstract

A 43-year-old male, known to be deaf, mute, and blind, presented to the ED accompanied by his daughter. She said that he had multiple episodes of diarrhea and vomiting for two days. At first, it gave the impression of gastroenteritis because the patient also had upper abdomen pain, but later a series of investigations were carried out, including blood tests, electrocardiogram (ECG), chest X-ray, ultrasound abdomen (USG), and CT of the kidney and the urinary bladder (CT KUB), to reach a diagnosis. It was confirmed on CT KUB that the patient had a stone in his ureter, which was causing dysuria and increased urinary frequency leading to multiple bathroom visits and vomiting due to severe pain. The patient was given analgesia, and the stone was removed via ureteroscopy. It was challenging to make a diagnosis due to a lack of communication, and it was an emotionally distressing case for the clinical team.

## Introduction

Abdominal pain can present many diseases in ED settings, such as acute pancreatitis, acute cholecystitis, myocardial infarction, gastroenteritis, etc. [1]. It is often a challenge to reach a definite diagnosis in such cases, and it can become more challenging if the patient cannot communicate properly just as in the case of a blind, deaf, and mute patient. It not only becomes a clinically confusing case but can also be emotionally distressing for the physician. As symptoms are not clearly described, and signs cannot be appropriately elicited, a series of investigations needs to be performed to rule out the cause of the patient’s presentation.

Renal or urinary tract stones usually present with severe renal colic associated with vomiting. They also lead to urinary tract infections (UTI), which leads to increased urinary frequency, dysuria, and urgency. These symptoms can be confused with various other diseases if the patient cannot give proper history, and treatment differs for every case. In the case of stones leading to renal colic, adequate analgesia along with medical or surgical treatment depending on the size of the stone is required [2]. This case report discusses a similar case to highlight the problems associated with dealing with a patient who is unable to communicate properly due to any disability.

## Case presentation

A 43-year-old male, known to be deaf, mute, and blind, presented to the ED accompanied by his daughter. She said that he had multiple episodes of diarrhea and vomiting for two days.

He was morbidly obese and diabetic, taking insulin. He had a history of coronary artery disease and had two coronary stents.

Taking more history asking about the presence of blood in the stool revealed that his daughter did not enter with him to the bathroom. As the patient was blind, deaf, and mute, diarrhea history also came under doubt at this point. What we could understand was that he had abdominal pain somewhere, vomited a few times, and entered the bathroom several times a day for two days.

He was vitally stable. Blood pressure was 130/76 mmHg, pulse was 92 beats per minute, respiratory rate was 16 breaths per minute, and the temperature was 37.3°C.

On examination, the patient was lying on the bed flat, looking well, morbidly obese. He was conscious, moving all limbs, had no rash, no neck rigidity. His chest was clear bilaterally, with no added sounds. His abdomen was lax with no localized tenderness, and hernia orifices were unremarkable. He was pointing to his epigastrium and right flank when asked for pain location. His extremities did not show redness, hotness, edema, or signs of deep vein thrombosis.

Investigations

Blood tests showed mild leukocytosis (WBCs 12000), mild anemia (Hb 11 g/dL), normal platelet count, mild elevation in kidney function (creatinine 120 mmol/L), slight elevation in liver function (alanine transaminase 70, aspartate transaminase 84), average bilirubin level, and mild elevation in amylase. Electrocardiogram (ECG) showed normal sinus rhythm and no signs of new ischemia. A chest X-ray showed no pneumonic patches or air under the diaphragm. The bedside ultrasound did not add so much as the patient was morbidly obese.

More blood tests, urine tests, and an official ultrasound (USG) for the abdomen were ordered. The lipase was normal. Troponin was negative.

The USG showed normal liver echotexture. The gall bladder wall thickness was average. The gall bladder showed no stones inside, and there were no signs of cholecystitis. The aorta was normal in caliber with no dilatation all over its course till the bifurcation. The left kidney had an average size with no hydronephrosis or stones. The right kidney showed moderate to severe hydronephrosis.

The urine analysis showed blood and mild leucocytes. So, the suspicion of renal colic became close in the picture of right moderate hydronephrosis, mild acute renal function derangement, and the blood in the urine.

**Figure 1:**
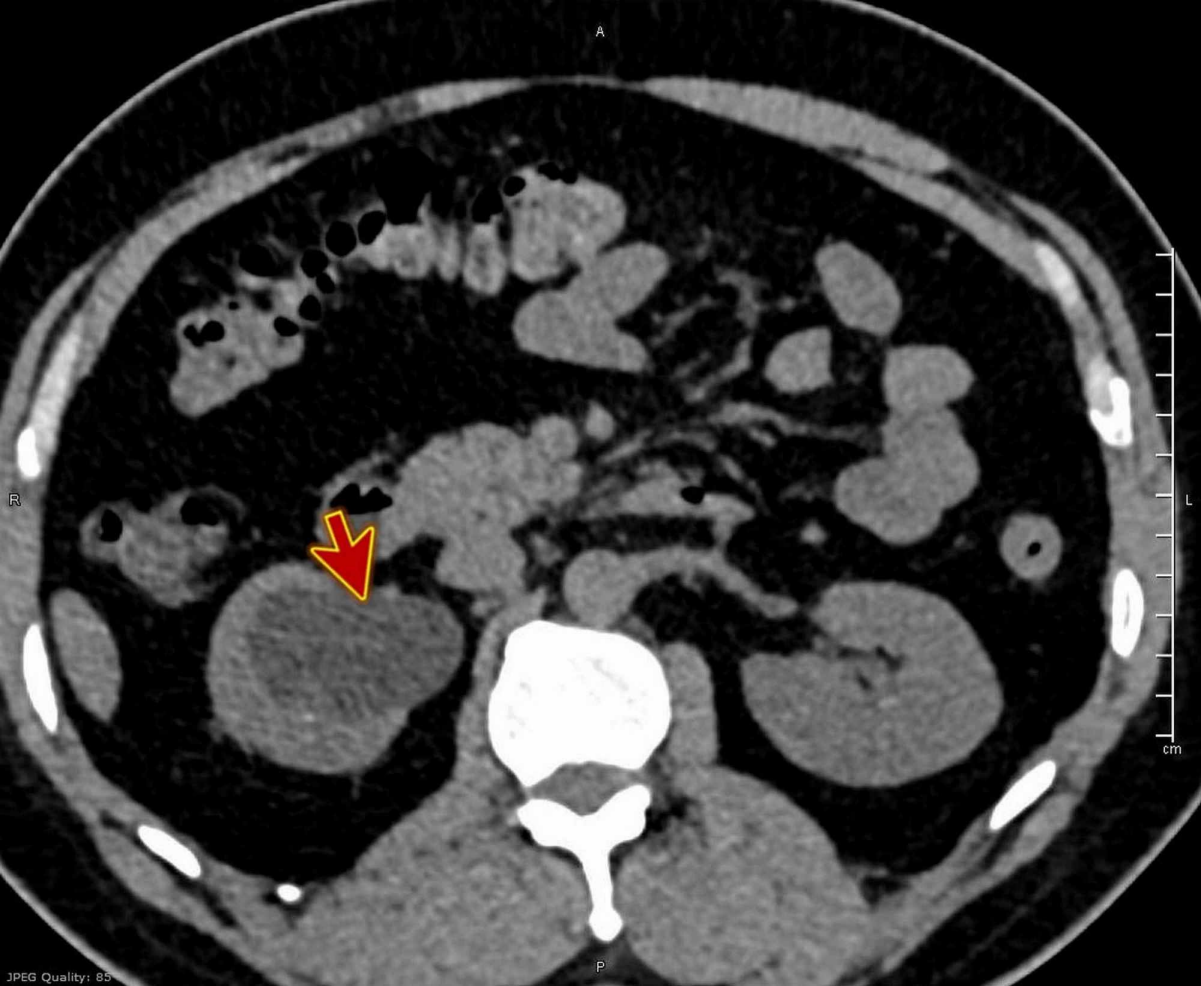
CT KUB showing right moderate to severe hydronephrosis (red arrow) and perinephric fat strandings. CT KUB, computed tomography of the kidney and the urinary bladder

**Figure 2:**
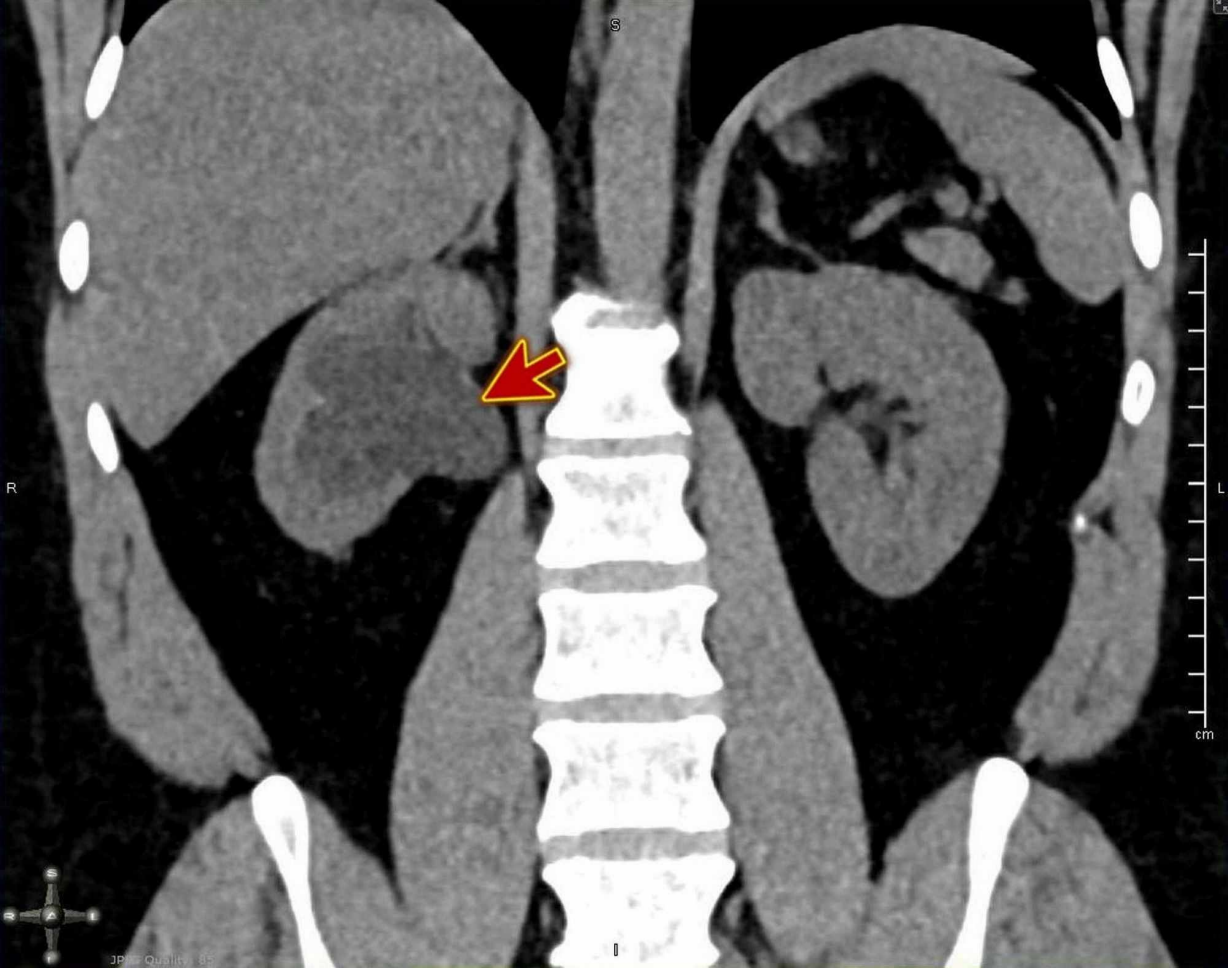
CT KUB showing right moderate to severe hydronephrosis (red arrow). CT KUB, computed tomography of the kidney and the urinary bladder

CT of the kidney and the urinary bladder (CT KUB) was ordered, which revealed a big stone 1.2 cm in the mid of the right ureter with right moderate to severe hydronephrosis and perinephric fat strandings (Figures 1-2).

Differential diagnosis

Many differential diagnoses were considered for a case of upper abdominal pain. The patient could have gastritis, gastroenteritis, acute pancreatitis, acute cholecystitis, renal colic, pneumonia, and acute myocardial infarction.

The lab investigations, in this case, helped rule out other differential diagnoses and reach a final diagnosis.

Ultrasound and blood tests ruled out gastritis and gastroenteritis. Lipase and amylase were normal, so the diagnosis of acute pancreatitis was out of consideration. There was a mild increase in liver enzymes, but no jaundice and no abnormality were seen in the liver and the gall bladder during USG, so the diagnosis of acute cholecystitis was also ruled out.

Chest X-ray was normal, so there was no question of pneumonia. Troponin and ECG also came out to be normal.

The USG showed hydronephrosis in the right kidney along with blood and WBCs in urinalysis. These findings prompted the suspicion of urinary tract stone, which was confirmed on CT KUB as it is the gold standard for the diagnosis of renal stones.

Treatment, outcome, and follow-up

The patient’s pain was controlled by simple analgesia. He was admitted to the urology department, where he underwent a ureteroscopy to remove the obstructing stone, and a Double-J (DJ stent) was inserted. The patient improved and was discharged home after two days without complications. He did not develop pain or hematuria. He followed with the urology department for three months.

Emotional challenges while dealing with this patient

The patient was in pain when he presented, but he could not communicate in any way because he was blind, mute, and deaf. His family communicated with him by touch. They used to hold his hands, draw some shapes, and point to some parts of his body. He was able to reply to his daughter’s touch with similar actions, and she delivered the translated message to the clinical team.

It was an emotionally overwhelming experience in multiple ways. Firstly, seeing how hard it was for the person to describe his problem and specifically pain at that time due to many disabilities was distressful. Secondly, we were not able to get the proper information from the patient, so it was hard to make differential diagnoses. There was a feeling of helplessness because we were not able to do something based on clinical findings. Even his daughter thought it was gastroenteritis because he went to the bathroom multiple times in the last two days. Later we figured out that it was because of dysuria and increased frequency of urination rather than diarrhea. Moreover, the patient was vomiting because of pain and not gastroenteritis.

## Discussion

Diarrhea, vomiting, and upper abdominal pain can be a presentation of various diseases, such as gastroenteritis, acute cholecystitis, and acute pancreatitis. When a patient presents in emergency settings with such symptoms, a clinician takes a detailed history and elicits signs to help reach a diagnosis [1]. It is a difficult job at times to reach a definite diagnosis in such presentations. However, it can become more complicated when the patient cannot communicate properly and can sometimes lead to a wrong diagnosis.

In this case, the patient was deaf, mute, and blind, which shows that communication was not possible. It was his family who communicated with him a bit through drawings and some signs. But it was not enough because there were high chances of miscommunication as also happened in this case. The patient’s daughter thought he had diarrhea, but that was not the real reason for frequent bathroom visits. Also, it is difficult to gain confidence in such patients while examining them. Such patients can get more anxious, and it can create a problem while examining. In one similar case, the patient endured a rectal tear during enema insertion. The patient was generally cooperative, but lack of communication increased the anxiety of the patient, which lead to such outcome [3]. Therefore, the communication gap not only causes a problem in history taking but also makes the examination difficult that can even lead to an incorrect diagnosis. A study found that poor communication can lead to a discontinuity of care, compromise of patient safety, and patient dissatisfaction [4]. It was highly possible in this case due to communication barriers. Even in cases where patients are deaf and mute, and sign language can be used, communication can be faulty. It can damage the autonomy of patients, limit access to services, and reduce the efficacy of treatment [5].

Difficult communication is not the only aspect of this case. The clinician who deals with such patients experiences various emotions. This case was an emotional experience for the doctor. It is a well-known fact that human interaction is associated with various feelings. It is not strange for a doctor to have emotions related to a patient in a clinical setting [6]. These emotional reactions can range from being mild to severe. Adverse patient-doctor interaction can lead to stress and even depression. They can result from daily experiences in clinical settings [7]. Taking care of disabled patients is more demanding in so many ways and needs special attention from the doctor. They are more vulnerable and need extra assistance [8]. This can be physically and emotionally distressing for a doctor too.

Unfortunately, extensive research has not been done on dealing with deaf, mute, and blind patients, limitations, and emotions related to it. There is a need to research this topic and provide comprehensive information.

## Conclusions

Deaf-mute-blind patients are too challenging in communication, especially in emergencies. Any vague history should not be trusted if it is obtained through a translator, and the differential diagnosis should be broadened in the situation of difficult communication; the diagnosis is by exclusion.
